# Efficacy and Mechanisms of CDK4/6 Inhibitors in Breast Cancer: Advancing Targeted Therapeutic Strategies

**DOI:** 10.32604/or.2026.073601

**Published:** 2026-03-23

**Authors:** Mohsina Patwekar, Faheem Patwekar, Zulhisyam Abdul Kari, Muhammad Rajaei Ahmad Mohd Zain, Arifullah Mohammed, Rohit Sharma

**Affiliations:** 1 Department of Agriculture Science, Faculty of Agro-Based Industry, Universiti Malaysia Kelantan, Jeli, Kelantan, Malaysia; 2 Department of Pharmacology, Luqman College of Pharmacy, PB 86, Old Jewargi Road, Gulbarga, Karnataka, India; 3 Department of Pharmacognosy, Luqman College of Pharmacy, PB 86, Old Jewargi Road, Gulbarga, Karnataka, India; 4 Advanced Livestock and Aquaculture Research Group, Faculty of Agro-Based Industry, Universiti Malaysia Kelantan, Jeli Campus, Jeli, Malaysia; 5 Department of Orthopaedics, School of Medical Sciences, Universiti Sains Malaysia, Kubang Kerian, Kelantan, Malaysia; 6 Department of Biotechnology, Koneru Lakshmaiah University (KLEF), Vaddeswaram Campus, Guntur, Andhra Pradesh, India; 7 Department of Rasa Shastra and Bhaishajya Kalpana, Faculty of Ayurveda, Institute of Medical Sciences, Banaras Hindu University, Varanasi, Uttar Pradesh, India

**Keywords:** Cyclin-dependent kinase 4/6 (CDK4/6) inhibitors, breast cancer, targeted therapy, endocrine therapy, resistance mechanisms, combination treatments

## Abstract

Breast cancer remains the primary cause of cancer-related mortality for women globally; therefore, further breakthroughs in treatment approaches are crucial. Palbociclib, ribociclib, and abemaciclib are among the Cyclin-dependent kinase 4 and 6 (CDK4/6) inhibitors that have become an innovative family of targeted therapy for hormone receptor-positive, Human Epidermal Growth factor receptor 2 (HR+/HER2−) breast cancer. These inhibitors work by preventing the action of CDK4/6, which are crucial in the regulation of the cell cycle. Leading cancer cells to cell cycle arrest and undergo apoptosis. When these inhibitors are used with endocrine medicines like letrozole and fulvestrant, clinical trials lead positive impact in progression-free survival and, in a few cases, complete survival. However, despite their effectiveness, resistance mechanisms are primary and current acquired problems, requiring combined approaches with additional targeted medicines and continuous investigation into innovative therapeutic plans. To maintain patient compliance and quality of life, common side effects such as tiredness, gastrointestinal problems, and neutropenia need to be effectively managed. There is hopefulness for wider oncological applications as next-generation CDK inhibitor development and adaptive clinical trials continue to test their potential beyond breast cancer. CDK4/6 inhibitors continue to be a key part of breast cancer treatment as cancer biology advances, marking a major advancement towards more potent and customized cancer medicines. This review aims to provide current evidence on CDK4/6 inhibitors in HR+/HER2− breast cancer, highlighting their mechanisms, interaction with endocrine resistance, combination strategies, and emerging biomarkers guiding personalized therapy.

## 1 Introduction

Breast cancer is the most common cancer in women across the globe to receive a diagnosis, which continues to be the main cause of mortality from cancer. The WHO (World Health Organization) predicts that breast cancer causes 2.3 million new cases and 685,000 deaths globally per year. Over the span of a lifetime, 1 in 8 American women is expected to be affected by breast cancer [1]. The GLOBOCAN 2020 report states that, with an estimated 2.3 million new cases (or 11.7% of all cancer diagnoses) and 685,000 deaths per year, breast cancer remains the most common cancer diagnosed globally. It is now the most frequently diagnosed cancer worldwide, surpassing lung cancer.

Changes in reproductive patterns are considered the main cause, with lifestyle factors, and enhanced screening programs, the frequency is higher in high-income and transitioning regions, such as North America, Western Europe, and parts of Asia. On the other hand, late-stage presentation and restricted access to healthcare continue to be significant hurdles in low and middle-income nations, where death ratios are disproportionately higher. Globally, the age-standardized mortality rate (ASMR) is 13.6 per 100,000 women, while the age-standardized incidence rate (ASIR) is roughly 47.8 per 100,000 women. Breast cancer accounts for almost 14% of female cancers in India alone, and its prevalence is steadily increasing in younger age groups. These numbers highlight the ongoing prevalence of breast cancer worldwide and the need for more individualized, accessible, and focused treatment approaches to enhance patient outcomes [1–3].

Current treatment modes for breast cancer are multifaceted and depend on subtypes of cancer, stage, and genetic characteristics. Often, the initial course of treatment, surgery involves either a mastectomy (removes the entire breast), or a lumpectomy (removes the lesion/tumor). Radiotherapy is used to reduce tumor size preoperatively or to eradicate any remnant post-surgery. Chemotherapy is a systemic approach that uses cytotoxic medications to destroy rapidly proliferating cells. Both before (neoadjuvant) and after (adjuvant) surgery, it can be used. Hormonal/Endocrine Therapy is a treatment that halts the body’s natural hormones like progesterone and estrogen from growing cancer by targeting hormone receptor-positive (HR+) breast cancers, e.g., Tamoxifen and aromatase inhibitors are common agents [2]. Targeted therapy in which medications that target the molecular alterations in cancer cells are selectively administered, examples include HER2-targeted medications such as pertuzumab and trastuzumab. Immunotherapy is a newly developed therapeutic strategy that boosts the immune system to identify and fight cancerous cells. Even with these medicines’ advances, there is no solution, especially with managing metastatic breast cancer and getting past resistance to existing treatments. Due to this, new treatment approaches have had to be developed, such as focusing on cell cycle regulators such as cyclin-dependent kinases (CDKs) [3].

CDKs are a class of serine/threonine kinases that are important for regulating the cell cycle. Cyclins, the regulatory subunits of CDKs, unite to create complexes that advance the cell cycle. Among them, CDK4 and CDK6 play a unique function in DNA replication when the G1 (first gap) phase transitions into the S (synthesis) phase [4]. In response to mitogenic signals, D-type cyclins (cyclins D1, D2 and D3) bind to CDK4/6. Cell Cycle Progression can be controlled by retinoblastoma (Rb) protein, an important tumor suppressor, which is phosphorylated by active cyclin D-CDK4/6 complexes. E2 Promoter-Binding Factor (Transcription Factor) (E2F) transcription factors are released by phosphorylated Rb and stimulate the transcription of genes required for DNA synthesis and S phase entrance [5].

Cyclin-dependent kinase inhibitors (CKIs), such as p16^INK4a, can bind to CDK4/6 and stop them from associating with cyclins, which inhibits the cell cycle itself. This tightly controls the activity of CDK4/6. A typical aspect of many malignancies, including breast cancer, is dysregulation of the CDK4/6-Rb-E2F pathway, which leads to unchecked cell proliferation [6]. Targeting CDK4/6 in breast cancer makes sense as kinases for controlling the cell cycle and tumor progression. Breast cancer regularly exhibits the abnormality within the CDK4/6 pathway, especially in HR+ subtypes, which frequently exhibit cyclin D1 overexpression or mutations that cause p16^INK4a to lose its activity [7]. HR+ Breast Cancer is typically responsible for about 70% of cases. These malignancies are sensitive to CDK4/6 inhibition because they majorly show dysregulation of the CDK4/6 pathway [8]. By specifically blocking CDK4/6 activity, drugs like palbociclib, ribociclib, and abemaciclib cause Rb to become hypo-phosphorylated, the G1 phase of the cell cycle to stop, and the proliferation of tumor cells to be inhibited. Endocrine treatment and CDK4/6 inhibitors together have shown synergistic activity that improves patient outcomes with HR+ breast cancer by postponing the course of illness. Two vital mechanisms that contribute to breast cancer’s development and survival are targeted by this combination [9]. All factors, CDK4/6 inhibitors are a huge step forward in the management of breast cancer, providing a focused strategy that targets the basic reason for tumor cell growth and resistance to traditional treatments [10]. This review aims to find out more about the mechanisms and effectiveness of these inhibitors in order to examine their potential as primary treatments for breast cancer.

## 2 Molecular Mechanisms of Action of CDK4/6 Inhibitors

### 2.1 The Cell Cycle and the Role of CDK4/6 in Cell Proliferation

**Figure 1:**
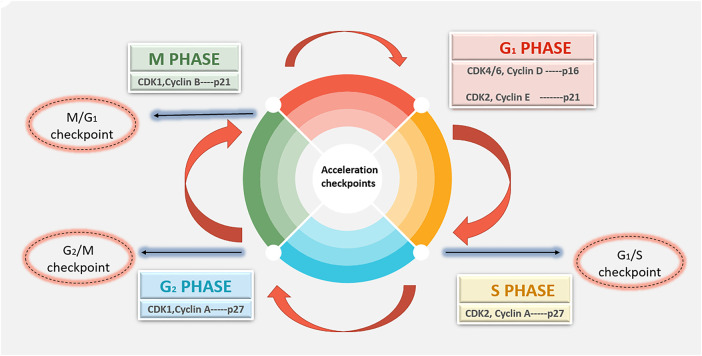
Schematic view of dysregulated cyclin-CDK signaling disrupts normal G_1_-M phase cell-cycle control, leading to uncontrolled proliferation in breast cancer. Abb: CDK—Cyclin-Dependent Kinase

A cell goes through a series of controlled phases known as the cell cycle in order to replicate and divide. A cell cycle deregulation in breast cancer can be found in Fig. 1.

The cell cycle is composed of 4 distinct phases, which include:

- **G1 Phase (First Gap):** During this phase, the cell divides and is ready for DNA synthesis.
- **S Phase (Synthesis):** Genetic material is doubled as a result of DNA replication.
- **G2 Phase (Second Gap):** During this phase, the cell keeps expanding and starts mitosis, making sure that all DNA is correctly duplicated and fixing any damage.
- **M Phase (Mitosis):** To create two daughter cells, the cell divides its cytoplasm and duplicate DNA.

The regulatory subunits of CDKs, cyclins, carefully regulate the movement between these phases. Particularly focusing on the G1 phase and the G1-to-S phase transition are CDK4 and CDK6 [5,11]. Activation of CDK4/6, in which D-type cyclins (cyclin D1, D2 and D3), which are communicated in response to external growth signals such as mitogens and growth factors, bind to CDK4 and CDK6 to activate them. The main tumor suppressor, Rb, is phosphorylated by the cyclin D-CDK4/6 complexes. The transcription of genes required for DNA synthesis and S-phase entrance is subsequently activated by the freeing of E2F transcription factors by phosphorylated Rb. CKIs, including p16^INK4a, control the activity of CDK4/6. These blockers have the capacity to attach to CDK4/6 and stop it from attaching to cyclins, which inhibits cyclin kinase activity and halts the progression of the cell cycle. A main feature is unchecked cell growth of many malignancies, including breast cancer. Dysregulation of the CDK4/6 pathway, such as overexpression of cyclin D1 or deletion of CKIs like p16^INK4a, causes this uncontrollable cell proliferation [4,5,11].

Although CDK4 and CDK6 have similar functions in promoting the G1 to S phase transition, new research indicates that they have different biological functions and are regulated differently in different tissues. The Rb is phosphorylated by both kinases when they create complexes with D-type cyclins (D1, D2 and D3). This results in the release of E2F transcription factors, which promote cell-cycle progression and proliferation. However, Cyclin D1, which is often overexpressed in breast, pancreatic, and colorectal cancers, strongly regulates CDK4, which is mainly expressed in epithelial and endocrine tissues. This CDK4-Cyclin D1 axis, which connects estrogen signalling to cell-cycle regulation, is essential in hormone receptor-positive breast cancer [5].

On the contrary, CDK6 is widely expressed in hematopoietic, mesenchymal, and neural progenitor cells, where it influences angiogenesis, cell differentiation, and immune response pathways in addition to helping to regulate the cell cycle. According to recent research, CDK6 may have kinase-independent roles that CDK4 does not, such as transcriptional control of VEGF-A and modulation of hematopoietic stem cell proliferation [4,5]. From a therapeutic point of view, the different tumor sensitivity to particular inhibitors may be explained by differential reliance on CDK4 versus CDK6. For example, abemaciclib shows a higher selectivity for CDK4, which contributes to its continuous dosing schedule and lower hematologic toxicity profile, while palbociclib and ribociclib show balanced inhibition of both kinases. It is essential to comprehend these minor but significant distinctions between CDK4 and CDK6 in order to predict resistance mechanisms in hormone receptor-positive breast cancer and optimize targeted therapy combinations [4,5,11].

### 2.2 Mechanisms by Which CDK4/6 Inhibitors Induce Cell Cycle Arrest and Apoptosis

Small compounds as CDK4/6 inhibitors, such as palbociclib, ribociclib, and abemaciclib, specifically block the kinase activity of CDK4 and CDK6, causing cell cycle arrest as well as apoptosis.

**Figure 2:**
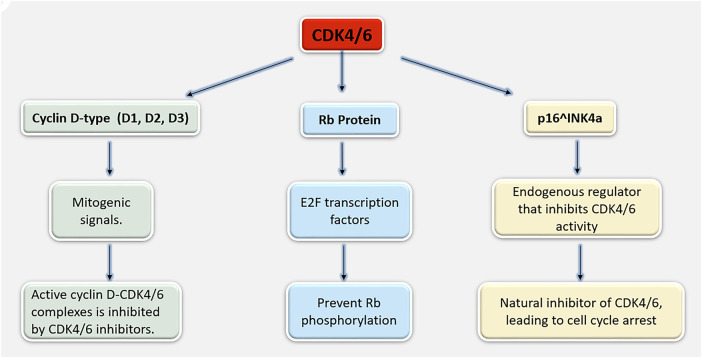
A mechanism illustrating cyclin D–CDK4/6, Rb, and p16^INK4a coordinate the G1–S cell-cycle transition and their regulation controls cell-cycle progression. Abb: D1, D2 and D3: D-type cyclins; E2F: E2 Promoter-Binding Factor

**Cell Cycle Arrest**: By reducing CDK4/6 activity, CDK4/6 inhibitors stop Rb from being phosphorylated. This means that Rb stays in its active, hypo-phosphorylated state, where it binds and sequesters E2F transcription factors. This causes cell cycle arrest in the G1 phase by inhibiting the transcription of E2F target genes, a basic requirement for DNA replication and S phase entry [12]. The G1/S checkpoint is a cell cycle mechanism that regulates the movement of cells from the G1 to the S phase (Fig. 2). The retinoblastoma protein and cyclin-dependent kinases CDK4/6 play key roles in this process.

**Induction of Apoptosis:** Apoptotic pathways can be triggered by prolonged cell cycle arrest. The regular cell cycle is upset when CDK4/6 is inhibited, which results in cellular stress and the activation of apoptotic signaling pathways. To further induce apoptosis in cancer cells, CDK4/6 inhibition may also upregulate pro-apoptotic molecules and downregulate anti-apoptotic proteins.

**Senescence:** Under a few conditions, suppression of CDK4/6 can result in senescence, a permanent cell cycle arrest in which cells maintain their metabolic activity but are not able to divide. Inflammatory cytokines and other substances that affect the tumor microenvironment are most commonly secreted by senescent cells [13,14].

### 2.3 Molecular Pathways Affected by CDK4/6 Inhibition

Additional to influencing the cell cycle, CDK4/6 inhibitors also affect a number of biochemical pathways associated with the development of cancer and resistance mechanisms:

PI3K/AKT/mTOR system is the signaling system is prime for metabolism, cell division, and survival. Through their interaction with this system, CDK4/6 inhibitors can affect the control of the cell cycle and lead to increased effectiveness of combined therapeutic approaches.

Signaling via the estrogen receptor (ER) often, endocrine therapy is used with CDK4/6 inhibitors in the treatment of HR+ breast cancer. Inhibiting CDK4/6 can make cancer cells more sensitive to estrogen deprivation and stop ER signaling-driven cell cycle progression [15]. DNA Damage Response (DDR) basically done by altering the production and action of DDR proteins, CDK4/6 inhibition can impede the DNA damage response. This increases the susceptibility of cells to substances that damage DNA and promote apoptosis. Immunological Modulation with high proof that CDK4/6 inhibitors alter the immunological milieu. By encouraging the infiltration and activation of immune cells, such as T cells, and reducing the immunosuppressive activity of regulatory T cells (Tregs), they can enhance anti-tumor immunity (Table 1). Generally, the capacity of CDK4/6 inhibitors to stop cell proliferation, trigger apoptosis, and alter several biochemical pathways that cause therapy resistance and cancer development makes them effective in breast cancer treatment. Majorly designed to target the basic processes governing cell cycle regulation, CDK4/6 inhibitors provide a highly effective and targeted means of treating breast cancer, especially in HR+ subtypes [16].

**Table 1:** Mechanisms of Cyclin-dependent kinase 4/6 (CDK4/6), the action of CDK4/6 inhibitors, and their effects on the cell cycle

Component	Mechanism	CDK4/6 inhibitor action	Effect on cell cycle
**CDK4/6**	Kinases dependent on cyclins attaching to D-type cyclins (D1, D2, D3) to generate functional complexes.	Inhibit kinase activity of CDK4/6 by binding to their ATP-binding sites.	Prevents phosphorylation of Rb protein.
**Cyclin D (D1, D2, D3)**	Regulatory subunits that bind to CDK4/6 in response to mitogenic signals.	The creation of active cyclin D-CDK4/6 complexes is inhibited by CDK4/6 inhibitors.	Active Rb continues to sequester E2F transcription factors.
**Rb Protein**	Tumor suppressor that regulates the cell cycle by binding to E2F transcription factors.	CDK4/6 inhibitors prevent Rb phosphorylation, maintaining it’s active, hypo phosphorylated state.	Cell cycle arrest at the G1 phase.
**E2F Transcription Factors**	Encourage the transcription of the genes needed for DNA synthesis and S phase entrance.	CDK4/6 inhibitors prevent the release of E2F from Rb, inhibiting transcription of S phase genes.	Inhibition of DNA synthesis and cell proliferation.
**p16^INK4a**	A cyclin-dependent kinase inhibitor that attaches itself to CDK4/6 and stops cyclin from binding.	Endogenous regulator that inhibits CDK4/6 activity.	Natural inhibitor of CDK4/6, leading to cell cycle arrest.
**Cell Cycle Phases**	G1, S, G2, M phases; regulated by cyclin-CDK complexes.	CDK4/6 inhibitors specifically target the G1-to-S phase transition.	Arrests cell cycle in the G1 phase, preventing DNA replication.

Note: **Abbreviation:** ATP: Adenosine Triphosphate; CDK4/6: Cyclin-Dependent Kinases 4 and 6; Cyclin D1/D2/D3: D-Type Cyclins (Regulatory Subunits); E2F: E2 Promoter-Binding Factor (Transcription Factor); G1 Phase: Gap 1 Phase (First Growth Phase of Cell Cycle); G2 Phase: Gap 2 Phase (Second Growth Phase before Mitosis); M Phase: Mitotic Phase; Rb: Retinoblastoma Protein; S Phase: Synthesis Phase (DNA Replication Phase).

**CDK4/6:** Central to the transition from G1 to S phase, used for cell proliferation.

**CDK4/6 Inhibitors:** Block CDK4/6 activity, prevent Rb phosphorylation, and maintain Rb in its active form.

**Effect on Cell Cycle:** Induces cell cycle arrest in the G1 phase, preventing the progression to DNA synthesis and subsequent cell division. These inhibitors can lead cancer cells to undergo cell cycle arrest, apoptosis, or even senescence, which reduces the growth and spread of tumors.

### 2.4 Epigenetic and Chromatin-Remodeling Alterations

Resistance to CDK4/6 inhibitors is increasingly understood to be largely caused by epigenetic and chromatin-remodeling changes. Despite CDK4/6 blockade, transcriptional reprogramming that encourages Cyclin E-CDK2 activation and cell-cycle progress is caused by dysregulation of chromatin modifiers like SWI/SNF complex subunits AT-Rich Interaction Domain 1A, SWI/SNF-Related, Matrix-Associated, Actin-Dependent Regulator of Chromatin Subfamily A Member 4 (ARID1A, SMARCA4), and over activity of Enhancer of Zeste Homolog 2 (EZH2), the catalytic component of Polycomb Repressive Complex 2 (PRC2). Lineage plasticity and upregulation of proliferative signalling pathways like PI3K/AKT/mTOR are further made possible by aberrant histone modifications, Trimethylation of Histone H3 on Lysine 27 (e.g., H3K27me3 gain), and DNA methylation changes. In order to overcome or postpone resistance, these reversible epigenetic changes offer a survival advantage under therapeutic pressure and point to novel combination strategies, such as combining CDK4/6 inhibitors with EZH2, Histone Deacetylase (HDAC), or Bromodomain and Extra-Terminal Motif (Protein Family) (BET) inhibitors [14,16].

### 2.5 Non-Coding RNAs (ncRNAs) and Metabolic Reprogramming

Resistance to CDK4/6 inhibitors is processed by metabolic reprogramming and non-coding RNAs (ncRNAs). Long non-coding RNAs (lncRNAs) and dysregulated microRNAs (miRNAs) can change the expression of important regulators like CDK6, Cyclin D1 Gene (CCND1), and RB1, which can result in persistent proliferation even after treatment. For example, increased CDK6 translation and Rb pathway evasion have been linked to downregulation of tumor-suppressive miR-29b and overexpression of lncRNA, Metastasis-Associated Lung Adenocarcinoma Transcript 1 (MALAT1) or HOX Transcript Antisense RNA (HOTAIR). At the same time, resistant cells show signs of metabolic adaptation, including increased lipid biosynthesis, mitochondrial oxidative phosphorylation, and glycolysis, that drive cell-cycle progression without any aid of CDK4/6 signalling. These results demonstrate the possibility of restoring therapeutic sensitivity and preventing adaptive resistance by targeting ncRNA-metabolic axes in addition to CDK4/6 inhibition [5,10,15].

## 3 Clinical Efficacy of CDK4/6 Inhibitors and Key Trail Outcomes

**Figure 3:**
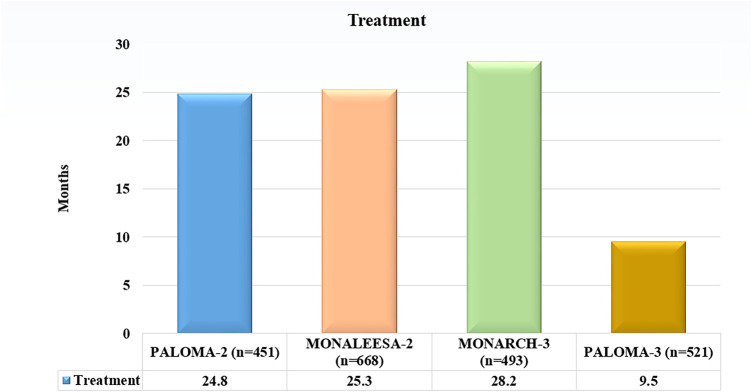
Comparative analysis of Median progression-free survival (PFS) across pivotal phase III trial evaluating CDK4/6 inhibitors combined with endocrine therapy in HR/HER2− breast cancer. Abb: HR+: Hormone Receptor-Positive, HER2−: Human Epidermal Growth factor receptor 2-Negative

The treatment of HR+, HER2-negative breast cancer has been improved because of CDK4/6 inhibitors. Clinical trials have examined three main CDK4/6 inhibitors in great detail: abemaciclib (Verzenio), palbociclib (Ibrance), and ribociclib (Kisqali) (Table 2). Although we describe briefly each Inhibitor below with key Clinical Trials, the panoramic view of CDK4/6 inhibitors sanctioned by the US Food and Drug Administration that have modified the treatment these days: palbociclib, ribociclib, and abemaciclib are shown in Fig. 3 (Table 3A,B).

**Table 2:** Comparative analysis of CDK4/6 inhibitors

Parameter	Palbociclib (Ibrance)	Ribociclib (Kisqali)	Abemaciclib (Verzenio)
**Median PFS (months)**	PALOMA-2: 24.8	MONALEESA-2: 25.3	MONARCH 3: 28.2
**Median OS (months)**	PALOMA-3: 34.9	MONALEESA-3: 53.7	MONARCH 2: 46.7
**Common side effects**	Neutropenia, fatigue, infections	Neutropenia, hepatotoxicity, QT prolongation	Diarrhea, neutropenia, fatigue
**Dose schedule**	21 days on, 7 days off	21 days on, 7 days off	Continuous daily dosing
**Combination therapies**	Letrozole, fulvestrant	Letrozole, fulvestrant, tamoxifen (premenopausal)	Aromatase inhibitors, fulvestrant

Note: **Abbreviation:** AI: Aromatase Inhibitor; FS: Follicle-Stimulating Hormone (if mentioned in hormonal context); HR+/HER2 Hormone Receptor-Positive/Human Epidermal Growth Factor Receptor 2-Negative; OS: Overall Survival; PFS: Progression-Free Survival; QT: Interval on Electrocardiogram Representing Ventricular Depolarization and Repolarization; SERM: Selective Estrogen Receptor Modulator (e.g., Tamoxifen); AE: Adverse Effect (for “Common Side Effects” if noted in legend); FDA: U.S. Food and Drug Administration (optional for drug approval context).

**Table 3:** (**A**) combination of CDK4/6 inhibitors with endocrine therapies. (**B**) combination of CDK4/6 inhibitors with targeted or immunotherapies

(A)				
Combination therapy	Clinical trial	Median progression-free survival (PFS)	Key findings	Common adverse effects
**Palbociclib + Letrozole**	PALOMA-2	24.8 months	Significant improvement in PFS compared with letrozole alone.	Neutropenia, fatigue, nausea
**Ribociclib + Letrozole**	MONALEESA-2	25.3 months	Marked increase in PFS vs. letrozole monotherapy.	Neutropenia, hepatotoxicity, QT prolongation
**Abemaciclib + Fulvestrant**	MONARCH 2	16.4 months	PFS significantly improved compared with fulvestrant alone.	Diarrhoea, fatigue, neutropenia
**Palbociclib + Fulvestrant**	PALOMA-3	9.5 months	Improved PFS relative to fulvestrant alone.	Neutropenia, fatigue, nausea
**Ribociclib + Fulvestrant**	MONALEESA-3	20.5 months	PFS significantly prolonged compared with fulvestrant alone.	Neutropenia, hepatotoxicity, QT prolongation
**Abemaciclib + Letrozole/Anastrozole**	MONARCH 3	28.2 months	Significant PFS enhancement compared with aromatase inhibitor monotherapy.	Diarrhoea, fatigue, neutropenia
(**B**)				
**Combination Therapy**	**Clinical Trial**	**Median Progression-Free Survival (PFS)**	**Key Findings**	**Common Adverse Effects**
**Palbociclib + Alpelisib (PI3K inhibitor)**	Ongoing clinical trials	Ongoing	Preclinical and early-phase studies show synergistic effects on tumor growth inhibition.	Awaiting mature safety data
**Ribociclib + Everolimus (mTOR inhibitor)**	Ongoing clinical trials	Ongoing	Preliminary data suggest potential enhancement of efficacy through mTOR pathway suppression.	Awaiting trial results
**Abemaciclib + Pembrolizumab (Immunotherapy)**	Ongoing clinical trials	Ongoing	Preclinical evidence indicates improved antitumor immune response and checkpoint modulation.	

Note: Abb: PI3K: Phosphatidylinositol 3-Kinase; mTOR: Mammalian Target of Rapamycin.

### 3.1 Palbociclib (Ibrance)

PALOMA-1/TRIO-18: This phase II trial examined the effects of palbociclib plus letrozole against letrozole alone in postmenopausal women with HR+/HER2HR+/HER2−advanced breast cancer. The combination resulted in high progression-free survival (PFS) from 10 months to 20 months approximately [17].

PALOMA-2: A phase III research that validated the findings of PALOMA-1, displaying that a median PFS of 24.8 months was achieved with palbociclib and letrozole in combination, as opposed to 14.5 months with letrozole alone. PALOMA-3: In phase III trial PALOMA-3, palombolib with fulvestrant was compared with placebo plus fulvestrant for patients with HR+/HER2−advanced breast cancer which increased after endocrine therapy. The median PFS, by contrast, was 4 months and 10 months approximately [18,19].

### 3.2 Ribociclib (Kisqali)

MONALEESA-2: In phase III trial, postmenopausal women with HR+/HER2−advanced breast cancer were chosen to examine the efficacy of letrozole alone against letrozole plus ribociclib. The median PFS for letrozole by itself was 16 months, but the combination was 25.3 months.

MONALEESA-3: In women who had reached menopause and had HR+/HER2−advanced breast cancer, ribociclib with fulvestrant was compared to placebo plus fulvestrant. The median PFS was 20.5 months as opposed to 12.8 months [20].

MONALEESA-7: Premenopausal women participated in a phase III trial comparing ribociclib plus goserelin and endocrine medication (either tamoxifen or a non-steroidal aromatase inhibitor) against a placebo plus goserelin and endocrine therapy. The median PFS was 23.8 months as opposed to 13.0 months [21].

### 3.3 Abemaciclib (Verzenio)

MONARCH 1: In a phase II trial, patients with HR+/HER2−metastasized breast cancer with unresponsive results to prior endocrine therapy or chemotherapy were chosen to evaluate abemaciclib as a monotherapy. The objective response rate (ORR) was 19.7%, and the median overall survival (OS) was 17.7 months [22].

MONARCH 2: In patients with HR+/HER2−advanced breast cancer progression prior endocrine therapy, a phase III trial with placebo plus fulvestrant to abemaciclib plus fulvestrant. The median PFS was 16.4 months as opposed to 9.3 months [23].

MONARCH 3: This phase III trial evaluated the effects of abemaciclib plus an aromatase inhibitor vs. placebo plus an aromatase inhibitor in patients with HR+/HER2−advanced breast cancer. The median PFS was 28.2 months as opposed to 14.8 months [24].

### 3.4 Case Studies and Real-World Evidence Supporting the Use of CDK4/6 Inhibitors

**Case Study 1:** Following progress on earlier endocrine treatment, a postmenopausal lady advanced HR+/HER2− positive breast cancer managed with palbociclib in conjunction with letrozole showed tumor shrinkage and prolonged PFS.

**Case Study 2:** This illustrates the effectiveness in younger patients: a premenopausal woman with HR+/HER2−metastasized breast cancer was able to attain clinical remission with ribociclib in addition to tamoxifen and goserelin [25].

**Case Study 3:** The effectiveness of abemaciclib monotherapy as a later-line treatment was exhibited by a patient whose breast cancer was advanced and HR+/HER2 extensive pretreatment [26].

### 3.5 Real-World Evidence

The efficacy of palbociclib outside of clinical trial settings was exhibited by a review of real-world data from the Flatiron Health database, which revealed that the average PFS for individuals receiving treatment with the drug was consistent with the outcomes of clinical trials [27].

**National Cancer Database (NCDB)**

Information from the NCDB supported the use of ribociclib in a variety of clinical settings by showing that patients’ outcomes were good compared to historical controls.

**Real-World Evaluation of Abemaciclib**

In a community oncology setting, a retrospective evaluation of abemaciclib produced acceptable toxicity status and PFS and OS that were equivalent to clinical trial results [28].

## 4 Combination Therapies Involving CDK4/6 Inhibitors

### 4.1 Exploration of Combination Strategies with Endocrine Therapies

When treating HR+/HER2− breast cancer, the combination of CDK4/6 inhibitors with endocrine treatments has proven to be the most successful. By simultaneously blocking hormone-driven and cell cycle pathways, these combinations increase therapeutic efficacy and postpone the onset of resistance. Key outcomes from clinical trials evaluating CDK4/6 inhibitors in combination with endocrine agents are summarized in Table 3A, while ongoing investigations involving targeted and immunotherapeutic combinations are presented in Table 3B [29].

#### 4.1.1 Palbociclib (Ibrance) with Letrozole

The PALOMA-2 Trial showed that PFS was considerably higher when palbociclib was added to letrozole than when letrozole was used alone. Comparing letrozole monotherapy to combination, the median PFS was 14.5 months, whereas it was 24.8 months. The aromatase inhibitor letrozole lowers estrogen levels, which in turn lowers signaling from the estrogen receptor (ER). Palbociclib amplifies this result by blocking CDK4/6, which stops the cell cycle [30].

#### 4.1.2 Ribociclib (Kisqali) with Letrozole

MONALEESA-2 Trial: indicates that ribociclib plus letrozole led to a median PFS of 25.3 months as opposed to letrozole alone, which produced a median PFS of 16 months. Ribociclib, like palbociclib, inhibits CDK4/6, which increases letrozole’s anti-proliferative actions [31].

#### 4.1.3 Abemaciclib (Verzenio) with Fulvestrant

MONARCH 2 Trial: found that abemaciclib plus fulvestrant significantly increased PFS (median PFS of 16.4 vs. 9.3 months) when compared to fulvestrant. By breaking down the ER, fulvestrant effectively lowers ER signaling. Abemaciclib produces a strong dual blockade by inhibiting CDK4/6, which further stops the progress of the cell cycle [32,33].

### 4.2 Synergistic Effects of Combining CDK4/6 Inhibitors with Other Targeted Therapies

Combining CDK4/6 inhibitors with other targeted therapies can potentially enhance anti-tumor effects and overcome resistance mechanisms.

#### 4.2.1 CDK4/6 Inhibitors with PI3K Inhibitors

The PI3K/AKT/mTOR pathway, which is important in cell survival and proliferation, is often activated in breast cancer. To increase anti-tumor effectiveness, CDK4/6 inhibitors and PI3K inhibitors jointly impede the pathways that contribute to cell cycle progression and survival. Preclinical Studies are being done in clinical settings to check the effectiveness of palbociclib in conjunction with the PI3K inhibitor alpelisib, as this combination has demonstrated potential [34,35].

#### 4.2.2 CDK4/6 Inhibitors with mTOR Inhibitors

Everolimus and other mTOR inhibitors block the mTOR pathway, which is important for cell growth and metabolism and lies downstream of PI3K/AKT. Inhibiting CDK4/6 and mTOR together can interfere with vital proliferative and survival signals. Preliminary efficacious studies combining ribociclib with everolimus and exemestane have been demonstrated; trials are underway to ascertain the most effective dosage [36,37].

### 4.3 Potential Biomarkers for Predicting Response to Combination Therapies

**Figure 4:**
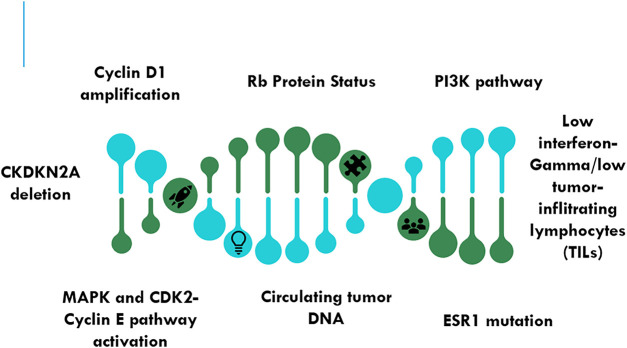
Emerging biomarkers for predicting response to combination therapies to CDK4/6 inhibitors. Abb: Rb—Retinoblastoma Protein; PI3K—Phosphatidylinositol-4,5-Bisphosphate 3-Kinase Catalytic Subunit Alpha; ESR1—Estrogen Receptor; MAPK—Mitogen-Activated Protein Kinase; CDKN2A—Cyclin-Dependent Kinase Inhibitor 2A

Various biomarkers to check CDK4/6 inhibitor-based combination therapies are crucial for personalizing treatment and improving outcomes (Fig. 4).

#### 4.3.1 Cyclin D1 Amplification

The mechanism of CDK4/6 pathway dependence is suggested by the overexpression of cyclin D1, a partner of CDK4/6. CDK4/6 drugs may work better for patients with cyclin D1 amplification. The clinical evidence indicates that patients who have amplification of cyclin D1 respond better to CDK4/6 inhibitors, according to retrospective analysis [38–40].

#### 4.3.2 Rb Protein Status

The Rb protein is the means by which CDK4/6 inhibitors work. Resistance to these inhibitors may be predicted by a decline in Rb function. Rb-positive tumors are more responsive to CDK4/6 inhibitors than Rb-negative tumors [41,42].

#### 4.3.3 PI3K Pathway Mutations

Alternative survival pathways may be triggered by mutations in the PI3K pathway, which may impact the body’s response to CDK4/6 inhibitors. In such circumstances, combining CDK4/6 inhibitors with inhibitors of the PI3K pathway may be especially beneficial. Trials that are still in progress are looking into how well PI3K pathway mutations predict a patient’s response to combo therapy [43,44].

#### 4.3.4 Estrogen Receptor Gene 1 (ESR1) Mutations

Resistance to endocrine therapy can result from mutations in the ESR1. The combination of CDK4/6 inhibitors and medicines aimed at ESR1 mutations improves the effectiveness of treatment. CDK4/6 inhibitors are beneficial when added to endocrine therapy for patients with ESR1 mutations, according to preliminary investigations [45].

To improve patient selection and track response to CDK4/6 inhibitors, large novel biomarkers are investigated in addition to well-established ones like Cyclin D1 amplification, intact Rb expression, PI3K pathway activation, and ESR1 mutations. Since loss of p16 or upregulation of Cyclin E can circumvent CDK4/6-Rb checkpoint control, Cyclin-Dependent Kinase Inhibitor 2A (CDKN2A) (p16^INK4A^) deletions and CCNE1 (Cyclin E1) amplifications have been associated with intrinsic resistance. Although rare at baseline, shortening mutations or RB1 loss often appear after extended therapy, which shows acquired resistance [38,39]. Nowadays, circulating tumor DNA (ctDNA) profiling provides a dynamic, non-invasive way to monitor these changes in real time. Weeks before radiologic progression, longitudinal ctDNA monitoring can identify early resistance events, such as mutations in FGFR1, KRAS, or ESR1, giving treatment adaptation a chance. Reduced sensitivity to CDK4/6 inhibition has also been predicted by phosphoproteomic signatures that show hyperactivation of the Mitogen-Activated Protein Kinase (MAPK) and CDK2-Cyclin E axes [40,42]. Overexpression of CDK7 and CDK9, which may drive compensatory transcriptional programs that sustain proliferation, and loss of FAT Atypical Cadherin 1(FAT1), which destabilizes Hippo-YAP (Yes-Associated Protein) signalling and confers resistance. Immune-related biomarkers, such as decreased tumor-infiltrating lymphocytes (TILs) and low interferon-gamma gene expression, are also highlighted by recent transcriptomic analyses. These biomarkers are associated with a decreased response to CDK4/6 inhibitors in conjunction with endocrine therapy [38–43]. Together, these developing biomarkers, which include immune, proteomic, and genomic aspects, are a new mode for precision-medicine options where molecular profiling directs the selection of CDK4/6 inhibitors, shows the onset of resistance, and helps in getting a good combination therapy.

### 4.4 Spatial and Transcriptomic Profiling

Single-cell RNA sequencing (scRNA-seq) and spatial transcriptomics are two examples of transcriptomic and spatial profiling technologies that focus on intratumoral heterogeneity and adaptive resistance to CDK4/6 inhibitors. Within HR+/HER2− breast tumours, these methods identify varied cellular subpopulations that differ in cell-cycle state, immune interactions, and pathway activation. PI3K/AKT pathway enrichment, Cyclin E1/CDK2 signalling, and E2F target gene upregulation are frequently observed in resistant clones, and spatial profiling identifies micro environmental slots that maintain proliferation in spite of treatment. Mapping drug-tolerant cell states and finding new transcriptional biomarkers that predict therapeutic response are made possible by integrating these datasets.

### 4.5 AI/ML and Radiomics Signatures

Emerging methods for forecasting CDK4/6 inhibitor response and resistance include radiomic analyses in conjunction with artificial intelligence (AI) and machine learning (ML) models. AI algorithms can find intricate biomarker signatures that are associated with treatment outcomes by combining multi-omic data, including genomic, transcriptomic, proteomic, and imaging features. It has been demonstrated that radiomic patterns from MRI or PET imaging provide non-invasive proxy images of molecular behaviour by reflecting underlying tumor proliferation and metabolic activity. In addition to improving patient stratification and treatment personalization, these computational models have the potential to detect resistance early and allow for dynamic, real-time therapeutic adjustments during CDK4/6-based therapy.

## 5 Resistance Mechanisms, Molecular Determinant, and Strategies to Overcome Resistance

### 5.1 Primary Resistance

This occurs when cancer cells inherent resistant to CDK4/6 inhibitors before treatment begins. Because CDK4/6 inhibitors work through the Rb protein, they may become ineffective if Rb is lost or mutated. Cyclin E can avoid the requirement for CDK4/6 activity by forming a complex with CDK2 to drive the G1 to S phase transition, which results in resistance. In spite of CDK4/6 inhibition, the cell cycle can continue due to elevated CDK2 levels that can make up for the inhibition of CDK4/6 [46,47].

### 5.2 Acquired Resistance

This develops after an initial response to CDK4/6 inhibitors. Acquired resistance can result from increased cyclin E1 levels, leading to cell cycle progression without the help of CDK4/6. Resistance arises from Rb gene deletions or secondary mutations that occur during therapy. Despite CDK4/6 suppression, cell survival and proliferation are enhanced by activating compensatory signaling pathways such as the PI3K/AKT/mTOR pathway. Cell cycle progression is sustained by increased CDK6 expression, which can counteract CDK4 inhibition [48,49].

### 5.3 Strategies to Overcome Resistance

Combining CDK4/6 inhibitors with other therapies can target multiple pathways and reduce the chances of resistance. By focusing on the PI3K/AKT/mTOR pathway and inhibiting CDK4/6, anti-tumor effectiveness is increased and resistance can be overcome. For instance, preclinical models have demonstrated synergistic effects when palbociclib and the PI3K inhibitor alpelisib are combined. Resistant usage of CDK4/6 inhibitors in conjunction with endocrine treatments like letrozole or fulvestrant can delay resistance. To further improve efficacy, new endocrine drugs such as selective estrogen receptor degraders (SERDs) may be added. By enhancing anti-tumor immunity and overcoming resistance mechanisms, immune checkpoint inhibitors (e.g., anti-PD-1 Programmed Cell Death Protein 1 /PD-L1 Programmed Death-Ligand 1 antibodies) may be useful with CDK4/6 inhibitors [50,51].

### 5.4 Novel Therapeutic Approaches

Developing new molecules and targeting emerging pathways can provide additional strategies to combat resistance. Since CDK2 is involved in avoiding CDK4/6 inhibition, it may be possible to overcome resistance by using selective CDK2 inhibitors. One of the main resistance mechanisms can be lessened by treatments that try to lower cyclin E levels or block its function. A more thorough reduction of cancer cell proliferation can be achieved by agents that inhibit multiple CDKs at once or combine CDK inhibition with other mechanisms (such as DNA damage response inhibitors) [52,53].

## 6 Ongoing Research and Future Directions in Overcoming Resistance

Identifying biomarkers that predict resistance can help tailor treatments and monitor resistance development. Early intervention is made possible by the real-time insights that ctDNA analysis can offer regarding genetic alterations and resistance mechanisms. Resistance mechanisms and possible targets for combination therapy can be found by examining changes in protein expression in response to CDK4/6 inhibitors [54,55]. These trials allow for modifications based on interim results, enabling the rapid evaluation of combination strategies and new agents. These trials evaluate a drug’s effectiveness in treating several types of cancer that have a similar molecular characteristic, speeding the discovery of potent therapies for breast cancer that are resistant to treatment [56]. Creating reliable preclinical models that faithfully replicate resistance mechanisms can help in the quest for new treatment approaches. Patient tumors are utilized to create Patient-Derived Xenografts (PDX) models, which can be used to evaluate novel treatment approaches and investigate resistance in a setting that is relevant to clinical practice [57]. In a more physiologically realistic setting, drug response and resistance mechanisms can be very well studied using breast cancer organoids, which offer a 3D culture system [58]. There is continuing research into next-generation CDK inhibitors that target more cyclins or have better potency and selectivity. These agents can potentially overcome resistance and offer more comprehensive inhibition of cell cycle progression by targeting different types of CDKs, like CDK1, CDK2, and CDK4/6. CDK9 is a newly discovered target that is involved in transcriptional control. When CDK4/6 inhibitors and CDK9 inhibitors are combined, cancer cell resistance and survival mechanisms may be affected [59].

## 7 Adverse Effects and Clinical Management

Despite their effectiveness, CDK4/6 inhibitors has lot of side effects that can affect patients’ quality of life and adherence to their treatment plans. The intensity and frequency of these side effects can fluctuate among the various CDK4/6 inhibitors (palbociclib, ribociclib, and abemaciclib). Lower neutrophil count, which raises the chance of infection. If required, supportive treatment with growth factors such as granulocyte-colony stimulating factor (G-CSF) is provided, along with routine blood count monitoring and dose modifications [60]. Often seen in individuals on CDK4/6 inhibitor therapy, there is a general sense of exhaustion or low vitality. Lifestyle changes include more rest, a healthy diet, and moderate exercise, addressing underlying conditions such as hypothyroidism or anemia. Diarrhea and Nausea with abemaciclib are very frequent. Digestion-related issues that may impact nutrition and hydration. Nutritional changes, hydration support, anti-diarrheal drugs (like loperamide), and anti-nausea drugs (like ondansetron).

The management of side effects linked to CDK4/6 inhibitors, especially diarrhea and neutropenia, necessitates a systematic approach that includes patient education, standardized dose modification protocols, and early detection, per the National Comprehensive Cancer Network (NCCN) (2024) and European Society for Medical Oncology (ESMO) (2023) clinical practice guidelines. It is advised to perform routine complete blood count (CBC) monitoring for hematologic toxicities every two weeks for the first two cycles, and then once a month after that. Instead of routinely using G-CSF in cases of grade 3 or 4 neutropenia, it is recommended to temporarily stop treatment and reduce dosage after recovery [60–63]. It is crucial to start antidiarrheal medications (like loperamide) as soon as possible and to stay properly hydrated in order to prevent gastrointestinal toxicities like diarrhoea, which is more frequently linked to abemaciclib. For persistent grade ≥2 symptoms, dose modifications or short-term cessation should be taken into consideration. In order to reduce unscheduled dose interruptions, NCCN and ESMO both stress the value of patient counselling prior to the therapy, with an emphasis on symptom recognition, adherence to monitoring plans, and timely reporting of side effects [64–67]. Hepatotoxicity is notable in ribociclib frequency. Increased liver enzymes signify injury to the liver. If severe, stop taking the medication, modify the dosage, or undergo regular liver function tests. Abstaining from alcohol and other hepatotoxic substances. Prolongation of the QT (Electrocardiographic Interval Representing Ventricular Depolarization and Repolarization) Interval is mainly linked to ribociclib. An increase in the heart’s QT interval length may cause arrhythmias. Monitoring of the electrocardiogram (ECG), dose modifications, avoidance of concurrent use of QT-prolonging medications, and electrolyte control. Venous Thromboembolism (VTE) with Abemaciclib was reported. Venous clots that can cause diseases such as pulmonary emboli or deep vein thrombosis. Early detection and treatment of VTE, prophylactic anticoagulation in high-risk patients, and symptom education for patients [62,63].

Management strategies to reduce adverse effects and improve patient quality of life is with Dose modifications. Toxicology can be controlled while preserving therapeutic efficiency by varying the dose of CDK4/6 inhibitors in accordance with the severity of adverse effects. Medication used to relieve symptoms and enhance patient comfort, such as growth factors, antiemetics, antidiarrheal, and analgesics. Early detection of undesirable effects enables prompt action. Laboratory tests (e.g., complete blood count, liver function tests, ECG) and clinical evaluations have to be performed on a regular basis. Patients can take charge of their treatment and prevent complications if they are informed about possible side effects, signs to look out for, and when to seek medical attention. Promoting a healthy lifestyle that includes frequent exercise, a balanced diet, and enough sleep might help reduce certain side effects and enhance general well-being [64,65].

## 8 Recent Studies and Trials of CDK4/6 Inhibitors in Breast Cancer

The safety, effectiveness, and wider applicability of CDK4/6 inhibitors in the treatment of breast cancer have been further investigated in recent trials and studies. Along with examining novel combination options and possible applications in additional cancer types, these trials also serve to uphold the advantages of currently available medicines (Table 4).

**Table 4:** Recent studies and trials of CDK4/6 inhibitors in breast cancer

Study/Trial	Phase	Combination therapy	Patient population	Key findings	Common adverse effects	Reference
MONARCH 2	III	Abemaciclib + Fulvestrant	HR+/HER2− advanced breast cancer, progressed on prior endocrine therapy	Significant improvement in PFS and OS compared to fulvestrant alone	Diarrhea, fatigue, neutropenia	[66]
MONARCH 3	III	Abemaciclib + Letrozole /Anastrozole	HR+/HER2− advanced breast cancer, first-line therapy	Significant improvement in PFS compared to letrozole/anastrozole alone	Diarrhea, fatigue, neutropenia	[67]
MONALEESA-3	III	Ribociclib + Fulvestrant	HR+/HER2− advanced breast cancer, both initial and after progression	Improved PFS and OS in both initial and after progression settings	Neutropenia, hepatotoxicity, QT prolongation	[66,67]
MONALEESA-7	III	Ribociclib + Endocrine Therapy + Goserelin	HR+/HER2− premenopausal women	PFS and OS were improved as compared to endocrine treatment alone.	Neutropenia, hepatotoxicity, QT prolongation	[68]
PALLAS	III	Palbociclib + Standard Adjuvant Endocrine Therapy	HR+/HER2− early breast cancer	Ongoing; preliminary data suggest potential benefits in PFS	Neutropenia, fatigue, infections	[68]
NATALEE	III	Ribociclib + Endocrine Therapy	HR+/HER2− early breast cancer	Ongoing; investigating adjuvant treatment benefits	Awaiting trial results	[69]
SOLAR-1	III	Alpelisib + Fulvestrant	HR+/HER2− advanced breast cancer, PIK3CA mutation	Significant improvement in PFS for patients with PIK3CA mutation	Hyperglycemia, rash, diarrhea	[69]

### 8.1 Trials for MONARCH 2 and 3

MONARCH 2 aimed to enroll women with HR+/HER2−advanced breast cancer who had not responded effectively to prior endocrine therapy. MONARCH 3 includes the first therapy for postmenopausal patients with HR+/HER2−advanced breast cancer. When compared to endocrine therapy alone, the outcomes of both trials showed that adding abemaciclib to endocrine therapy significantly increased both OS and PFS. Diarrhea, tiredness, and neutropenia were common side effects; patients receiving abemaciclib were more likely to experience diarrhea [70,71].

### 8.2 Trial Design and Population for MONALEESA-3 and 7

MONALEESA-3 comprised postmenopausal women with HR+/HER2−advanced breast cancer who were receiving fulvestrant and ribociclib as initial treatment, as well as following endocrine therapy progression. MONALEESA-7 evaluated ribociclib in conjunction with goserelin and endocrine therapy with a focus on premenopausal women. Both studies showed significant increases in PFS and OS, proving that ribociclib is a useful treatment for a range of patient categories. Notable side effects that needed to be regularly monitored and managed were neutropenia, hepatotoxicity, and QT interval prolongation [72,73].

### 8.3 PALLAS Trial Design and Population

In patients with early-stage HR+/HER2− breast cancer, the PALLAS trial examined the addition of palbociclib to conventional adjuvant endocrine therapy. The trial is still in progress, but early results point to possible improvements in PFS. Fatigue, neutropenia, and an elevated risk of infections were among the frequent side effects [74].

### 8.4 NATALEE Study Design and Population

The NATALEE study is assessing the effectiveness of ribociclib as a complementary therapy for HR+/HER2− early breast cancer when combined with endocrine therapy. The research is still in progress, and it is anticipated that the results will highlight the benefits of ribociclib when used as an adjuvant [75].

### 8.5 Population and Design of the SOLAR-1 Trial

The SOLAR-1 trial evaluated fulvestrant plus alpelisib, a PI3K inhibitor, in patients with HR+/HER2−advanced breast cancer that carried a Phosphatidylinositol-4,5-Bisphosphate 3-Kinase Catalytic Subunit Alpha (PIK3CA) mutation. PFS for patients with the PIK3CA mutation was considerably enhanced by the combination. Hyperglycemia, dermatitis, and diarrhea were frequent side effects [76].

## 9 Future Perspectives

In an effort to maximize benefits, minimize side effects, and lower resistance, new CDK4/6 inhibitors are being developed. Dalpiciclib, a CDK4/6 inhibitor, is now being evaluated in clinical trials to determine its efficacy and safety in HR+/HER2− breast cancer. Trilaciclib is a medication that may be used in conjunction with CDK4/6 inhibitors due to its propensity to preserve bone marrow and lessen myelosuppression brought on by chemotherapy. They selectively inhibit CDK4/6 or target several CDKs, potentially providing more thorough cell cycle inhibition with fewer adverse effects [77,78]. Possibility of CDK4/6 Inhibitors in Other Cancer Types, although HR+/HER2− breast cancer is the main indication for CDK4/6 inhibitors, further research is investigating their potential in other cancer types. For non-small cell lung cancer (NSCLC), trials are examining the use of CDK4/6 inhibitors in conjunction with immunotherapies or targeted treatments. Preclinical research indicates that CDK4/6 inhibitors improves glioblastoma treatment outcomes, namely the effectiveness of chemotherapy and radiotherapy. For melanoma with particular genetic alterations, CDK4/6 inhibitors are being tested in conjunction with v-Raf Murine Sarcoma Viral Oncogene Homolog B1 (BRAF) and Mitogen-Activated Protein Kinase (MEK) inhibitors. To overcome resistance in pancreatic cancer, research is investigating the use of CDK4/6 inhibitors in combination with other targeted medicines [79,80].

## 10 Limitations

Even while CDK4/6 inhibitors show promise in treating HR+/HER2− breast cancer, there are still a number of drawbacks. Primary and acquired resistance are a major obstacle that lowers these medicines’ long-term efficacy. Research is needed to create effective counterstrategies because mechanisms of resistance, like loss of Rb protein, overexpression of cyclin E, and activation of alternative signaling pathways, require constant attention [81]. Furthermore, the side effects of CDK4/6 inhibitors, including as hepatotoxicity, neutropenia, and QT interval prolongation, might lower the adherence to therapy and negatively affect the level of the patient’s wellbeing. The disturbances in patient reactions emphasize the necessity for predictive biomarkers in order to better customize therapies. Furthermore, these inhibitors’ cost may prevent them from being accessible, particularly in environments with limited resources. Lastly, although CDK4/6 inhibitors exhibit potential outside of breast cancer, more clinical research is necessary to see whether these drugs are effective in treating other cancer types. To optimize the therapeutic potential of CDK4/6 inhibitors and enhance results for a larger patient group, these constraints must be addressed [82].

## 11 Conclusion

In certain situations, the overall survival of patience, CDK4/6 inhibitors have revolutionized treatment approaches in hormone receptor-positive, HER2-negative breast cancer. These inhibitors, in particular palbociclib, ribociclib, and abemaciclib, majorly inhibit tumor growth and improve the effectiveness of endocrine treatments like letrozole and fulvestrant by particularly preventing the cell cycle’s transition from the G1 to the S phase. They act as standard care medicine in both first-line and refractory mode has been strongly established by data from clinical trials such as PALOMA, MONALEESA, and MONARCH. With the introduction of CDK4/6 inhibitors, the management of breast cancer has reformed from being primarily endocrine-based towards biologically informed and targeted, improving disease control and quality of life for patients with advanced phases of cancer.

However, problems persist in the form of acquired and primary resistance, which is frequently brought on by changes in the Retinoblastoma (Rb) pathway, Cyclin E amplification, or alternative signalling pathway activation. Current work aims to overcome these constraints by developing next-generation CDK inhibitors, PI3K and mTOR inhibitor combination therapies, and biomarker-driven methods for resistance monitoring and prediction. Although gastrointestinal problems, fatigue, and neutropenia are still more common side effects, tolerability has increased due to dose management and patient education. Future directions focus on expanding into other cancer types and incorporating immune checkpoint inhibitors, epigenetic modulators, and adaptive trial designs. All of these initiatives highlight how CDK4/6 inhibition is still developing for precision oncology, with the potential to provide more potent, customized, and long-lasting cancer treatments.

## Data Availability

The data that support the findings of this study are available from the Corresponding Author, [Zulhisyam Abdul Kari], upon reasonable request.

## Abbreviations

AI: Artificial Intelligence
AKT: Protein Kinase B
ARID1A: AT-Rich Interaction Domain 1A
ASIR: Age-Standardized Incidence Rate
ASMR: Age-Standardized Mortality Rate
ATAC-seq: Assay for Transposase-Accessible Chromatin using Sequencing
BAF: BRG1/BRM-Associated Factor (SWI/SNF complex)
BET: Bromodomain and Extra-Terminal Motif Protein
CCNE1: Cyclin E1
CDK: Cyclin-Dependent Kinase
CDK4/6: Cyclin-Dependent Kinases 4 and 6
CDKN2A: Cyclin-Dependent Kinase Inhibitor 2A (p16^INK4A^)
CI: Confidence Interval
ctDNA: Circulating Tumor DNA
CUT&RUN: Cleavage Under Targets and Release Using Nuclease
E2F: E2 Promoter-Binding Factor
EGFR: Epidermal Growth Factor Receptor
ER: Estrogen Receptor
ESMO: European Society for Medical Oncology
ESR1: Estrogen Receptor 1 Gene
EZH2: Enhancer of Zeste Homolog 2
FDA: U.S. Food and Drug Administration
FGFR1: Fibroblast Growth Factor Receptor 1
G-CSF: Granulocyte-Colony Stimulating Factor
GLOBOCAN: Global Cancer Observatory Database
HDAC: Histone Deacetylase
HER2: Human Epidermal Growth Factor Receptor 2
HOTAIR: HOX Transcript Antisense RNA
HR: Hormone Receptor
HR+/HER2−: Hormone Receptor-Positive, HER2-Negative
IFN-γ: Interferon-Gamma
lncRNA: Long Non-Coding RNA
MAPK: Mitogen-Activated Protein Kinase
MALAT1: Metastasis-Associated Lung Adenocarcinoma Transcript 1
miRNA: MicroRNA
ML: Machine Learning
mTOR: Mammalian Target of Rapamycin
MRI: Magnetic Resonance Imaging
NCCN: National Comprehensive Cancer Network
ncRNA: Non-Coding RNA
ORR: Objective Response Rate
PALOMA: Palbociclib Clinical Trial Series
PFS: Progression-Free Survival
PET: Positron Emission Tomography
PI3K: Phosphatidylinositol 3-Kinase
PI3K/AKT/mTOR: Phosphoinositide 3-Kinase/Protein Kinase B/Mammalian Target of Rapamycin Pathway
PR: Progesterone Receptor
PRC2: Polycomb Repressive Complex 2
Rb: Retinoblastoma Protein
RNA: Ribonucleic Acid
scRNA-seq: Single-Cell RNA Sequencing
SCS: Spinal Cord Stimulation
SMARCA4: SWI/SNF-Related, Matrix-Associated, Actin-Dependent Regulator of Chromatin Subfamily A Member 4
SWI/SNF: SWItch/Sucrose Non-Fermentable (Chromatin Remodeling Complex)
TENS: Transcutaneous Electrical Nerve Stimulation
TILs: Tumor-Infiltrating Lymphocytes
VEGF-A: Vascular Endothelial Growth Factor A
WHO: World Health Organization
